# Advances in Understanding Hair Growth

**DOI:** 10.12688/f1000research.7520.1

**Published:** 2016-02-08

**Authors:** Bruno A. Bernard

**Affiliations:** 1 L’Oréal Research and Innovation, Asnières-sur-Seine, France

**Keywords:** Glyco-biology, hair follicle biology, alopecia, hair cycling, glycan

## Abstract

In this short review, I introduce an integrated vision of human hair follicle behavior and describe opposing influences that control hair follicle homeostasis, from morphogenesis to hair cycling. The interdependence and complementary roles of these influences allow us to propose that the hair follicle is a true paradigm of a “Yin Yang” type, that is a cold/slow-hot/fast duality. Moreover, a new promising field is emerging, suggesting that glycans are key elements of hair follicle growth control.

## Introduction

The hair follicle is a true paradigm of mesenchymal-epithelial interaction. From early morphogenesis to a fully formed organ, the hair follicle life-cycle is controlled by a dialog between mesenchymal and epithelial compartments ^1^. However, this dialog relies on a delicate balance between conflicting and/or opposing influences.

**Figure 1.**
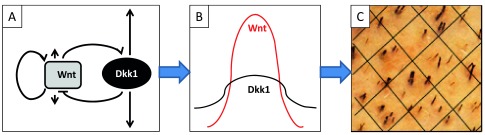
From reaction-diffusion to hair follicle patterning. ( **A**) Wnt morphogen stimulates its own synthesis as well as that of Dkk1, its inhibitor. Wnt diffuses slowly while Dkk1 diffuses rapidly. ( **B**) As a result, in a periodic way, Wnt concentration is higher than that of DKK1, and a hair placode can develop. ( **C**) The reaction-diffusion process thus explains the patterned distribution of hair follicles at the surface of the scalp.

With respect to hair follicle morphogenesis, the reaction-diffusion model explains how slowly diffusing inducers and rapidly diffusing inhibitors orchestrate, through local activation and at distance inhibition, the hair follicle patterned formation. Indeed, the seminal work of A. Turing ^2^ has been recently confirmed through a formal identification of morphogen activator-inhibitor couples, such as Wnt/DKK1 ^3^ ( Figure 1) and EDAR/BMP ^4^.

Considering its dual mesenchymal and epithelial origin, the hair follicle can be considered a composite organ, with a concentric structure. Dermal and epithelial compartments interact with each other and are characterized by specific differentiation programs. Opposing signaling pathways concur to control the unique behavior of human hair follicle and maintain its unique intrinsic homeostasis. As the activity of diffusible factors, such as growth factors and morphogens, can be modulated by glycans, their possible role in hair growth control must be taken into account.

## Hair follicle behavior

**Figure 2.**
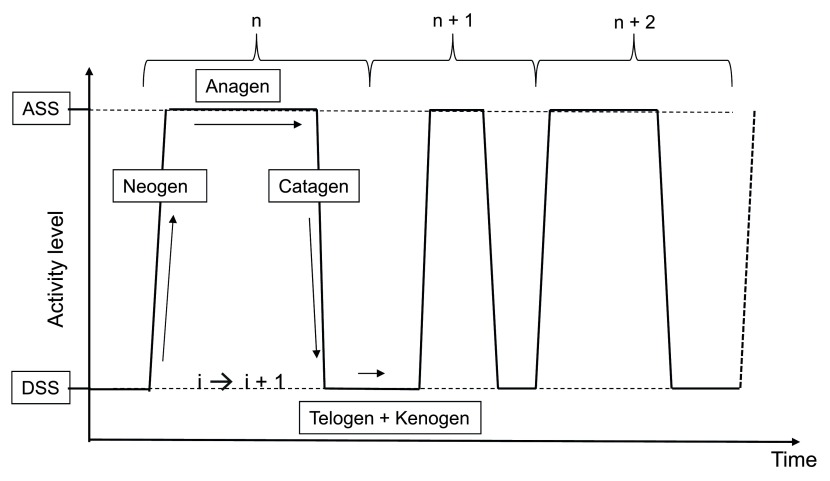
New representation of hair follicle behavior. An active steady state (ASS) of fiber production (anagen) and a dormant steady state (DSS) (telogen/kenogen) are interspaced by short-lasting phases of regression (catagen) and neomorphogenesis (neogen).

The hair follicle is the only organ in mammals that sequentially and repeatedly transits from a phase of active fiber production (anagen) to a resting phase (telogen), through rapid phases of tissue regression (catagen) and regeneration (neogen). A recently published comprehensive guide describes most of the morphological and immunohistological markers that characterize the different stages of the human hair follicle cycle and the intense tissue remodeling events which take place ^5^. Of note, hair follicle regeneration relies on the cyclical activation of stem cells ^6^. In the human hair follicle, these stem cells are harbored within two distinct reservoirs ^7, 8^, one of them bathing in a hypoxic environment ^9^. Instead of a cyclical behavior with an intrinsic automaton, the human hair follicle exhibits a stochastic behavior, the probability of duration of each phase fitting with a lognormal equation ^10^. A new concept ( Figure 2) postulates the existence of a bi-stable equilibrium ^11^ which controls human hair follicle dynamics, including an active steady state (the anagen stage) and a resting steady state (the telogen stage), the transition between these two steady states involving either a degradation phase (the catagen phase) or a neo-morphogenesis phase (the neogen phase). It is now believed that mesenchymal and epithelial oscillators control the stochastic autonomous switching between these two steady states ^12, 13^. The transition phases are both controlled by a complex and dynamic network of interacting activators and inhibitors, diffusible morphogens, and growth factors of opposite influences ^14^. Of note, however, extrapolating from results only obtained in rodents must be approached with caution, since major differences exist between human and mouse hair follicles in terms of phase duration, synchronicity, tissue remodeling, stem cell reservoirs, and so on.

**Figure 3.**
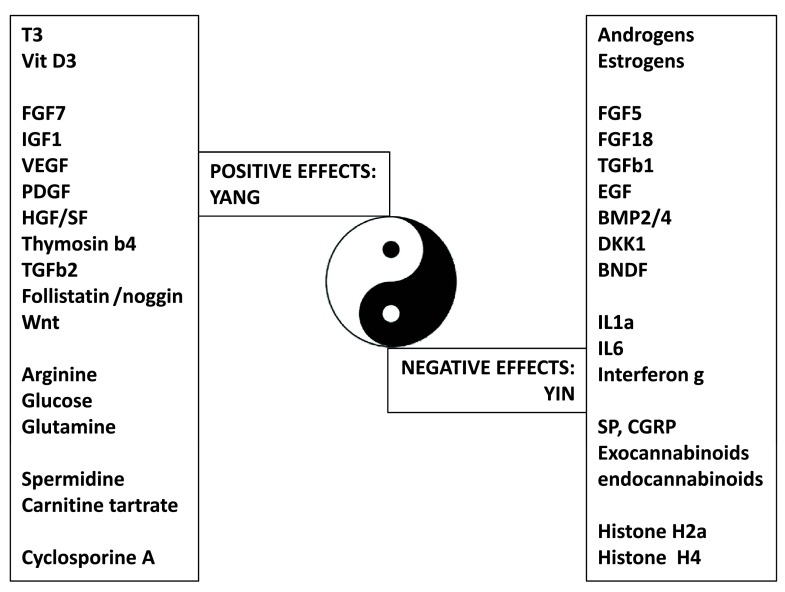
The Yin Yang of the human hair follicle. Summary of diffusible factors having positive (Yang) or negative (Yin) effects on hair growth and cycling.

During the active steady state, hair fiber production results from a finely, timely, and spatially tuned choreography of gene expression, which is highly sensitive to stimulatory and inhibitory signals. A number of signaling pathways ^15^, cytokines ^16, 17^, neuropeptides ^18^, hormones ^19– 22^, prostaglandins ^23^, and growth factors ^24^ are known to modulate the duration of the active steady state of the hair follicle ( Figure 3). For example, while insulin-like growth factor (IGF)-1 is required for anagen maintenance ^25, 26^, fibroblast growth factor (FGF)-5 appears to be a crucial regulator of hair length in humans ^27^, as a strong inducer of the catagen phase. Moreover, the human hair follicle is endowed with an autonomous androgen metabolism ^28^, a strict dependence on arginine ^29^, polyamines ^30^, and glucose ^31^ for growth, and a specific immunological response ^32^. The hair follicle is also endowed with a full prostaglandin metabolism and a complex network of prostaglandin (PG) receptors ^33, 34^. Recent data suggest that a delicate equilibrium between PGE2/PGF2a on the one hand and PGD2 on the other hand controls the duration of the active steady state. PGE2/PGF2a promotes hair growth maintenance, while PGD2 inhibits it and triggers anagen to catagen transition ^35^. Finally, re-evaluating the mechanisms by which agents such as cyclosporine A ^36^ or JAK-STAT inhibitors ^37^ promote human hair growth might help to identify new key genes and pathways involved in the control of hair growth.

Besides the active steady state, new data demonstrate that the resting steady state is not as quiescent as suspected and can be divided into a refractory period and a permissive period. Indeed, during the telogen phase, the follicle is under the influence of factors that would repress the onset of the neogen phase and factors that would trigger it. Specifically, a strong expression of bone morphogenetic protein (BMP) and FGF-18 defines the refractory period, during which the neogen onset is prevented. The progressive increase in the production of BMP antagonist noggin, Wnt/Fzz/b-catenin pathway activators, and transforming growth factor (TGF)-β2 then reaches a critical threshold that shifts the telogen follicle to a competency status, receptive to FGF-7, secreted by the nearby dermal papilla, and, ultimately, triggers the onset of the neogen phase ^38^.

## Glyco-biology of the human hair follicle

It is clear from the above that the complex and rhythmic behavior of the human hair follicle is under the control of multiple, intricate pathways with opposing influences. In this respect, the interdependence and complementary roles of these influences allow us to propose that the hair follicle is a true paradigm of a “Yin Yang” type duality and harmony. However, in our opinion, the fine tuning of these influences cannot solely rely on the timely and spatially controlled gene expression, but also on glycans, “the third revolution in evolution” ^39^. Glycans are endowed with such a huge molecular diversity that they can be considered the third language of life, after DNA and proteins.

**Figure 4.**
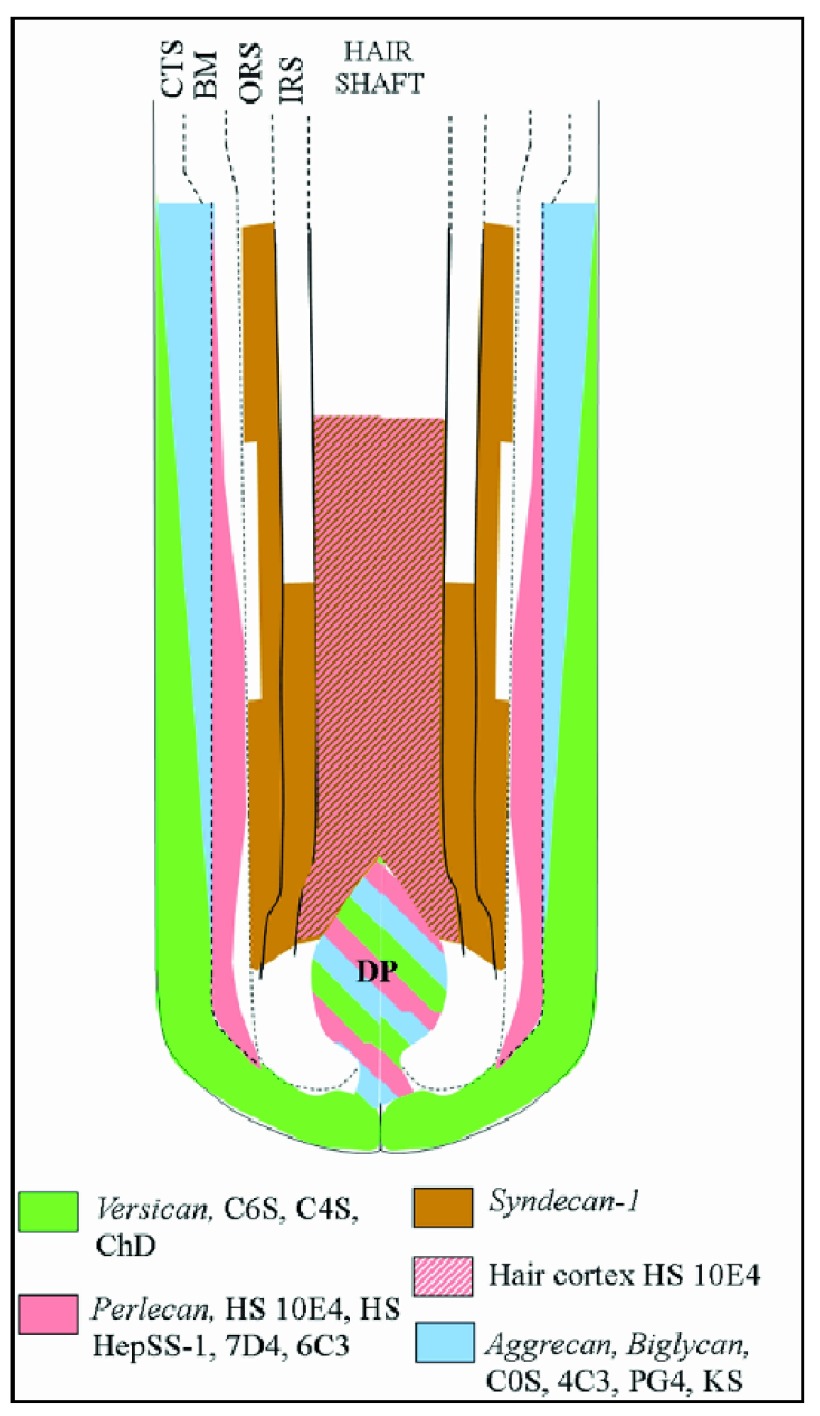
Diagram of proteoglycan expression in the human hair follicle. Diagram shows the distribution of versican, perlecan, syndecan 1, aggrecan, biglycan, and heparan sulfate proteoglycans in the different hair follicle compartments. BM, basement membrane; CTS, connective tissue sheath; IRS, inner root sheath; ORS, outer root sheath.

**Figure 5.**
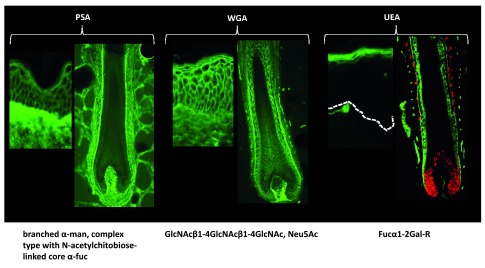
Diagram of proteoglycan expression in the human hair follicle. Distribution of N-glycans identified by their reactivity with fluorescently labelled *Pisum sativum* agglutinin (PSA), wheat germ agglutinin (WGA) and *Ulex europeus* agglutinin (UEA) in both skin and hair follicles. PSA mainly decorates the dermal compartments of skin and hair follicles, while WGA decorates both dermal and epithelial compartments. UEA only decorates the epidermis stratum granulosum and the hair follicle IRS.

Linear or branched oligosaccharides can be attached to a protein backbone *via* O-(serine/threonine) or N-(asparagine) linkages. They form the large class of N-Complex type glycans. Glycosaminoglycans are linear copolymers of 6-O-sulfated disaccharide units which define them as chondroitin, dermatan, keratin, or heparin sulfates. Proteoglycans have one or more glycosaminoglycan side chains attached to a core protein. Glycosaminoglycans, proteoglycans, and glycan moieties of glycoproteins have long been known to play important roles in the maintenance of protein conformation and solubility, protection against proteolytic degradation, mediation of biological activity, intracellular sorting and externalization, and embryonic development and differentiation ^40– 45^. The distribution of proteoglycans in the human hair follicle was originally described in the early 1990s, namely for chondroitin sulfate, dermatan sulfate, and heparin sulfate proteoglycans ^46^, for syndecan 1, perlecan and decorin ^47^, and for versican ^48^. Thanks to the availability of new immunological tools, the distribution of proteoglycans in the human hair follicle has been further refined ^49^ ( Figure 4), highlighting a complex, dynamic, and regionalized network of proteoglycans. With respect to cell surface complex type N-glycans, the use of specific fluorescently labeled lectins (saccharide-binding proteins) revealed a differential N-glycan composition among the different hair follicle compartments ^50– 52^ ( Figure 5).

What could be the role of these glycans? It has been known for quite a long time that growth factor activation could be regulated by proteoglycans ^53, 54^ and that heparan sulfate proteoglycans were involved in fine-tuning mammalian physiology ^55^ and in cell signaling during development ^56^. With respect to key regulators of hair follicle growth and cycling, syndecans modulate Wnt signaling cascades ^57^, the glycosaminoglycan chains of proteoglycans shape Hedgehog gradients and signal transduction ^58^, and O-linked glycosylation controls Notch1 interaction with its cognate Delta-like 4 receptor ^59^. Decorin, a small leucine-rich proteoglycan, directly modulates TGF-β, epidermal growth factor (EGF), IGF-1 and hepatocyte growth factor (HGF) signaling, all known actors of hair follicle cycling ^60^, and appears to act as an anagen inducer ^61^. Altogether, these recent results designate glycans as long time ignored key players in hair growth control. But, on top of that, enzymes can further modulate the biological activity of these glycans. For example, fucosyl transferase is absolutely required for Notch activity, and disruption of fucosyl transferase expression in murine hair follicle lineages results in aberrant telogen morphology, a decrease of bulge stem cell markers, a delay in anagen re-entry, and dysregulation of proliferation and apoptosis during the hair cycle transition ^62^. With respect to proteoglycans, heparanase (an endoglycosidase that cleaves heparin sulfate) was found expressed in the outer root sheath of murine hair follicles and identified as an important regulator of hair growth through its ability to release heparin-bound growth factors ^63^. In the human hair follicle, however, heparanase was found located in the inner root sheath. Its inhibition provoked an immediate transition from anagen to catagen ^64^. In this case, the HPSG/heparanase network appears to be a key controller of internal hair follicle homeostasis.

Finally, extracellular sulfatases appear to be critical regulators of heparin sulfate activities. Sulf1 and Sulf2, by removing glucosamine-6S groups from specific regions of heparan sulfate chain, modulate (a) Wnt interaction with its cognate receptor Frizzled, (b) BMP signaling by releasing BMP antagonist Noggin, and (c) FGF-2 ability to form the functional FGF-2-HS-FGFR ternary complex ^65, 66^. Of note, TGF-β1, by inducing Sulf1 expression ^67^, might indirectly modulate Wnt, BMP, and FGF-2 activities, which could explain its inhibitory effect on hair growth. From a clinical point of view, alterations of glycosaminoglycan degradation provoke mucopolysaccharidoses and abnormalities in hair morphology ^68^, which can be reversed by appropriate enzyme replacement therapy ^69^.

## Conclusion

The hair follicle is clearly endowed with a unique behavior. Its bi-stability and the intense remodeling processes that it provokes rely on the permanent dialog between opposing and complementary influences, impacting all follicle compartments. From this interdependent duality, one can easily understand that an optimal way to describe the complex equilibrium which controls hair follicle homeostasis is the concept of “Yin Yang”. Until recently, the understanding of hair growth mainly relied on deciphering the patterns of gene expression within the different hair follicle compartments throughout the hair cycle ^70, 71^. From now on, the fine-tuning of the activities of growth factors and morphogens by the modulating effects of glycans will also have to be taken into consideration.

From a prospective point of view, it is likely that a better understanding of hair diseases, and more specifically the role of inflammation and immune response in the development of alopecia areata ^72^ and androgenetic alopecia ^73^, will likely provide further insights into the role of the so-called immune privilege ^74^ in hair growth control. Moreover, with the advent of mature metabolomics technologies ^75^ coupled with *in vitro* human hair growth technology ^76^, one can predict that this integrative approach will permit us to identify these key metabolic pathways sustaining normal hair growth.

## References

[1] Sennett R Rendl M: Mesenchymal-epithelial interactions during hair follicle morphogenesis and cycling. *Semin Cell Dev Biol.* 2012;23(8):917–927. 10.1016/j.semcdb.2012.08.011 22960356 PMC3496047

[2] Turing A: The chemical basis of morphogenesis. *Philos Trans R Soc Lond B Biol Sci.* 1952;237:37–72. 10.1098/rstb.1952.0012 PMC4360114 25750229

[3] Schlake T Sick S: Canonical WNT signalling controls hair follicle spacing. *Cell Adh Migr.* 2007;1(3):149–151. 10.4161/cam.1.3.5073 19262137 PMC2634019

[4] Mou C Jackson B Schneider P: Generation of the primary hair follicle pattern. *Proc Natl Acad Sci U S A.* 2006;103(24):9075–9080. 10.1073/pnas.0600825103 16769906 PMC1482568

[5] Oh JW Kloepper J Langan EA: A guide to Studying Human Hair Follicle Cycling *In Vivo*. *J Invest Dermatol.* 2015;136(1):34–44. 10.1038/jid.2015.354 PMC4785090 26763421

[6] Alonso L Fuchs E: The hair cycle. *J Cell Sci.* 2006;119(Pt 3):391–393. 16443746 10.1242/jcs.02793

[7] Commo S Gaillard O Bernard BA: The human hair follicle contains two distinct K19 positive compartments in the outer root sheath: a unifying hypothesis for stem cell reservoir? *Differentiation.* 2000;66(4–5):157–164. 10.1046/j.1432-0436.2000.660401.x 11269941

[8] Purba TS Haslam IS Poblet E: Human epithelial hair follicle stem cells and their progeny: current state of knowledge, the widening gap in translational research and future challenges. *Bioessays.* 2014;36(5):513–525. 10.1002/bies.201300166 24665045

[9] Rathman-Josserand M Genty G Lecardonnel J: Human hair follicle stem/progenitor cells express hypoxia markers. *J Invest Dermatol.* 2013;133(8):2094–2097. 10.1038/jid.2013.113 23474947

[10] Halloy J Bernard BA Loussouarn G: Modeling the dynamics of human hair cycles by a follicular automaton. *Proc Natl Acad Sci U S A.* 2000;97(15):8328–8333. 10.1073/pnas.97.15.8328 10899998 PMC26947

[11] Bernard BA: The human hair follicle, a bistable organ? *Exp Dermatol.* 2012;6(8):401–403. 10.1111/j.1600-0625.2012.01457.x 22458655

[12] Al-Nuaimi Y Goodfellow M Paus R: A prototypic mathematical model of the human hair cycle. *J Theor Biol.* 2012;310:143–159. 10.1016/j.jtbi.2012.05.027 22677396

[13] Tasseff R Bheda-Malge A DiColandrea T: Mouse hair cycle expression dynamics modeled as coupled mesenchymal and epithelial oscillators. *PLoS Comput Biol.* 2014;11(8):e1003914. 10.1371/journal.pcbi.1003914 25375120 PMC4222602

[14] Murray PJ Maini PK Plikus MV: Modelling hair follicle growth dynamics as an excitable medium. *PLoS Comput Biol.* 2012;8(12):e1002804. 10.1371/journal.pcbi.1002804 23284275 PMC3527291

[15] Lee J Tumbar T: Hairy tale of signaling in hair follicle development and cycling. *Semin Cell Dev Biol.* 2012;23(8):906–916. 10.1016/j.semcdb.2012.08.003 22939761 PMC3496046

[16] Mahé YF Buan B Billoni N: Pro-inflammatory cytokine cascade in human plucked hair. *Skin Pharmacol.* 1996;9(6):366–375. 10.1159/000211447 9055357

[17] Kwack MH Ahn JS Kim MK: Dihydrotestosterone-inducible IL-6 inhibits elongation of human hair shafts by suppressing matrix cell proliferation and promotes regression of hair follicles in mice. *J Invest Dermatol.* 2012;132(1):43–49. 10.1038/jid.2011.274 21881585

[18] Samuelov L Kinori M Bertolini M: Neural controls of human hair growth: calcitonin gene-related peptide (CGRP) induces catagen. *J Dermatol Sci.* 2012;67(2):153–155. 10.1016/j.jdermsci.2012.04.006 22677443

[19] Billoni N Buan B Gautier B: Thyroid hormone receptor beta1 is expressed in the human hair follicle. *Br J Dermatol.* 2000;142(4):645–652. 10.1046/j.1365-2133.2000.03408.x 10792213

[20] Meier N Langan D Hilbig H: Thymic peptides differentially modulate human hair follicle growth. *J Invest Dermatol.* 2012;132(5):1516–1519. 10.1038/jid.2012.2 22402437

[21] Inui S Itami S: Molecular basis of androgenetic alopecia: From androgen to paracrine mediators through dermal papilla. *J Dermatol Sci.* 2011;61(1):1–6. 10.1016/j.jdermsci.2010.10.015 21167691

[22] Hu HM Zhang SB Lei XH: Estrogen leads to reversible hair cycle retardation through inducing premature catagen and maintaining telogen. *PLoS One.* 2012;7(7):e40124. 10.1371/journal.pone.0040124 22792225 PMC3390338

[23] Khidhir KG Woodward DF Farjo NP: The prostamide-related glaucoma therapy, bimatoprost, offers a novel approach for treating scalp alopecias. *FASEB J.* 2013;27(2):557–567. 10.1096/fj.12-218156 23104985 PMC3545535

[24] Imamura T: Physiological functions and underlying mechanisms of fibroblast growth factor (FGF) family members: recent findings and implications for their pharmacological application. *Biol Pharm Bull.* 2014;37(7):1081–1089. 10.1248/bpb.b14-00265 24988999

[25] Philpott MP Sanders DA Kealey T: Effects of insulin and insulin-like growth factors on cultured human hair follicles: IGF-I at physiologic concentrations is an important regulator of hair follicle growth *in vitro*. *J Invest Dermatol.* 1994;102(6):857–861. 8006448 10.1111/1523-1747.ep12382494

[26] Ahn SY Pi LQ Hwang ST: Effect of IGF-I on Hair Growth Is Related to the Anti-Apoptotic Effect of IGF-I and Up-Regulation of PDGF-A and PDGF-B. *Ann Dermatol.* 2012;24(1):26–31. 10.5021/ad.2012.24.1.26 22363152 PMC3283847

[27] Higgins CA Petukhova L Harel S: FGF5 is a crucial regulator of hair length in humans. *Proc Natl Acad Sci U S A.* 2014;111(29):10648–10653. 10.1073/pnas.1402862111 24989505 PMC4115575

[28] Gerst C Dalko M Pichaud P: Type-1 steroid 5 alpha-reductase is functionally active in the hair follicle as evidenced by new selective inhibitors of either type-1 or type-2 human steroid 5 alpha-reductase. *Exp Dermatol.* 2002;11(1):52–58. 10.1034/j.1600-0625.2002.110106.x 11962492

[29] Michelet JF Bernard BA Juchaux F: Importance of L-Arginine for human hair growth. *28th IFSCC Meeting Proceedings.* 2014;1123–1128.

[30] Ramot Y Marzani B Pinto D: N ^1^-methylspermidine, a stable spermidine analog, prolongs anagen and regulates epithelial stem cell functions in human hair follicles. *Arch Dermatol Res.* 2015;307(9):841–847. 10.1007/s00403-015-1592-9 26216444

[31] Williams R Philpott MP Kealey T: Metabolism of freshly isolated human hair follicles capable of hair elongation: a glutaminolytic, aerobic glycolytic tissue. *J Invest Dermatol.* 1993;100(6):834–840. 8496624 10.1111/1523-1747.ep12476744

[32] Paus R Nickoloff BJ Ito T: A 'hairy' privilege. *Trends Immunol.* 2005;26(1):32–40. 10.1016/j.it.2004.09.014 15629407

[33] Colombe L Vindrios A Michelet JF: Prostaglandin metabolism in human hair follicle. *Exp Dermatol.* 2007;16(9):762–769. 10.1111/j.1600-0625.2007.00586.x 17697149

[34] Colombe L Michelet JF Bernard BA: Prostanoid receptors in anagen human hair follicles. *Exp Dermatol.* 2008;17(1):63–72. 10.1111/j.1600-0625.2007.00639.x 18005048

[35] Garza LA Liu Y Yang Z: Prostaglandin D _2_ inhibits hair growth and is elevated in bald scalp of men with androgenetic alopecia. *Sci Transl Med.* 2012;4(126):126ra34. 10.1126/scitranslmed.3003122 22440736 PMC3319975

[36] Hawkshaw NJ Haslam IS Ansell DM: Re-Evaluating Cyclosporine A as a Hair Growth-Promoting Agent in Human Scalp Hair Follicles. *J Invest Dermatol.* 2015;135(8):2129–2132. 10.1038/jid.2015.121 25826423

[37] Harel S Higgins CA Cerise JE: Pharmacologic inhibition of JAK-STAT signaling promotes hair growth. *Sci Adv.* 2015;1(9):e1500973. 10.1126/sciadv.1500973 26601320 PMC4646834

[38] Geyfman M Plikus MV Treffeisen E: Resting no more: re-defining telogen, the maintenance stage of the hair growth cycle. *Biol Rev Camb Philos Soc.* 2015;90(4):1179–1196. 10.1111/brv.12151 25410793 PMC4437968

[39] Lauc G Krištić J Zoldoš V: Glycans - the third revolution in evolution. *Front Genet.* 2014;5:145. 10.3389/fgene.2014.00145 24904645 PMC4033155

[40] Boucaut JC Bernard B Aubery M: Concanavalin A binding to amphibian embryo and effect on morphogenesis. *J Embryol Exp Morphol.* 1979;51:63–72. 383873

[41] Bernard BA Yamada KM Olden K: Carbohydrates selectively protect a specific domain of fibronectin against proteases. *J Biol Chem.* 1982;257(14):8549–8554. 7045125

[42] Codogno P Bernard B Font J: Changes in protein glycosylation during chick embryo development. *Biochim Biophys Acta.* 1983;763(3):265–275. 10.1016/0167-4889(83)90134-9 6626582

[43] Bernard BA Newton SA Olden K: Effect of size and location of the oligosaccharide chain on protease degradation of bovine pancreatic ribonuclease. *J Biol Chem.* 1983;258(20):12198–12202. 6630185

[44] Olden K Bernard BA Humphries M: Function of glycoprotein glycans. *TIBS.* 1985;10(2):78–82. 10.1016/0968-0004(85)90238-5

[45] Wang H Zhou T Peng J: Distinct roles of *N*-glycosylation at different sites of corin in cell membrane targeting and ectodomain shedding. *J Biol Chem.* 2015;290(3):1654–1663. 10.1074/jbc.M114.606442 25451932 PMC4340409

[46] Westgate GE Messenger AG Watson LP: Distribution of proteoglycans during the hair growth cycle in human skin. *J Invest Dermatol.* 1991;96(2):191–195. 1704038 10.1111/1523-1747.ep12461019

[47] Couchman JR: Hair follicle proteoglycans. *J Invest Dermatol.* 1993;101(1 Suppl):60S-64. 10.1111/1523-1747.ep12362642 8326155

[48] du Cros DL LeBaron RG Couchman JR: Association of versican with dermal matrices and its potential role in hair follicle development and cycling. *J Invest Dermatol.* 1995;105(3):426–431. 10.1111/1523-1747.ep12321131 7665924

[49] Malgouries S Thibaut S Bernard BA: Proteoglycan expression patterns in human hair follicle. *Br J Dermatol.* 2008;158(2):234–242. 10.1111/j.1365-2133.2007.08339.x 18067481

[50] Ohno J Fukuyama K Epstein WL: Glycoconjugate expression of cells of human anagen hair follicles during keratinization. *Anat Rec.* 1990;228(1):1–6. 10.1002/ar.1092280102 1700646

[51] Tezuka M Ito M Ito K: Differential analysis of the human anagen hair apparatus using lectin binding histochemistry. *Arch Dermatol Res.* 1991;283(3):180–185. 10.1007/BF00372059 1867481

[52] Heng MC Levine S Fine H: Expression of the L-fucose moiety on infrainfundibular follicular keratinocytes of terminal follicles, its decreased expression on vellus and indeterminate follicles of androgenetic alopecia, and re-expression in drug-induced hair regrowth. *J Invest Dermatol.* 1992;98(1):73–78. 1370232 10.1111/1523-1747.ep12495536

[53] Schlessinger J Lax I Lemmon M: Regulation of growth factor activation by proteoglycans: what is the role of the low affinity receptors? *Cell.* 1995;83(3):357–360. 10.1016/0092-8674(95)90112-4 8521464

[54] Kresse H Schönherr E: Proteoglycans of the extracellular matrix and growth control. *J Cell Physiol.* 2001;189(3):266–274. 10.1002/jcp.10030 11748584

[55] Bishop JR Schuksz M Esko JD: Heparan sulphate proteoglycans fine-tune mammalian physiology. *Nature.* 2007;446(7139):1030–1037. 10.1038/nature05817 17460664

[56] Lin X: Functions of heparan sulfate proteoglycans in cell signaling during development. *Development.* 2004;131(24):6009–6021. 10.1242/dev.01522 15563523

[57] Pataki CA Couchman JR Brábek J: Wnt Signaling Cascades and the Roles of Syndecan Proteoglycans. *J Histochem Cytochem.* 2015;63(7):465–480. 10.1369/0022155415586961 25910817

[58] Whalen DM Malinauskas T Gilbert RJ: Structural insights into proteoglycan-shaped Hedgehog signaling. *Proc Natl Acad Sci U S A.* 2013;110(41):16420–16425. 10.1073/pnas.1310097110 24062467 PMC3799379

[59] Luca VC Jude KM Pierce NW: Structural biology. Structural basis for Notch1 engagement of Delta-like 4. *Science.* 2015;347(6224):847–853. 10.1126/science.1261093 25700513 PMC4445638

[60] Inui S Itami S: A newly discovered linkage between proteoglycans and hair biology: decorin acts as an anagen inducer. *Exp Dermatol.* 2014;23(8):547–548. 10.1111/exd.12471 24942290

[61] Jing J Wu XJ Li YL: Expression of decorin throughout the murine hair follicle cycle: hair cycle dependence and anagen phase prolongation. *Exp Dermatol.* 2014;23(7):486–491. 10.1111/exd.12441 24816226

[62] Lin HY Kao CH Lin KM: Notch signaling regulates late-stage epidermal differentiation and maintains postnatal hair cycle homeostasis. *PLoS One.* 2011;6(1):e15842. 10.1371/journal.pone.0015842 21267458 PMC3022660

[63] Zcharia E Philp D Edovitsky E: Heparanase regulates murine hair growth. *Am J Pathol.* 2005;166(4):999–1008. 10.1016/S0002-9440(10)62321-8 15793281 PMC1602387

[64] Malgouries S Donovan M Thibaut S: Heparanase 1: a key participant of inner root sheath differentiation program and hair follicle homeostasis. *Exp Dermatol.* 2008;17(12):1017–1023. 10.1111/j.1600-0625.2008.00739.x 18557927

[65] Lamanna WC Kalus I Padva M: The heparanome--the enigma of encoding and decoding heparan sulfate sulfation. *J Biotechnol.* 2007;129(2):290–307. 10.1016/j.jbiotec.2007.01.022 17337080

[66] Seffouh A Milz F Przybylski C: HSulf sulfatases catalyze processive and oriented 6- *O*-desulfation of heparan sulfate that differentially regulates fibroblast growth factor activity. *FASEB J.* 2013;27(6):2431–2439. 10.1096/fj.12-226373 23457216

[67] Yue X Li X Nguyen HT: Transforming growth factor-beta1 induces heparan sulfate 6- *O*-endosulfatase 1 expression *in vitro* and *in vivo*. *J Biol Chem.* 2008;283(29):20397–20407. 10.1074/jbc.M802850200 18503048 PMC2459296

[68] Malinowska M Jakóbkiewicz-Banecka J Kloska A: Abnormalities in the hair morphology of patients with some but not all types of mucopolysaccharidoses. *Eur J Pediatr.* 2008;167(2):203–209. 10.1007/s00431-007-0462-7 17361416

[69] Kloska A Bohdanowicz J Konopa G: Changes in hair morphology of mucopolysaccharidosis I patients treated with recombinant human alpha-L-iduronidase (laronidase, Aldurazyme). *Am J Med Genet A.* 2005;139(3):199–203. 10.1002/ajmg.a.31021 16283671

[70] Ohyama M Kobayashi T Sasaki T: Restoration of the intrinsic properties of human dermal papilla *in vitro*. *J Cell Sci.* 2012;125(Pt 17):4114–4125. 10.1242/jcs.105700 22623722

[71] Sennett R Wang Z Rezza A: An Integrated Transcriptome Atlas of Embryonic Hair Follicle Progenitors, Their Niche, and the Developing Skin. *Dev Cell.* 2015;34(5):577–591. 10.1016/j.devcel.2015.06.023 26256211 PMC4573840

[72] Kang H Wu WY Lo BK: Hair follicles from alopecia areata patients exhibit alterations in immune privilege-associated gene expression in advance of hair loss. *J Invest Dermatol.* 2010;130(11):2677–2680. 10.1038/jid.2010.180 20613773

[73] Mahé YF Michelet JF Billoni N: Androgenetic alopecia and microinflammation. *Int J Dermatol.* 2000;39(8):576–584. 10.1046/j.1365-4362.2000.00612.x 10971723

[74] Christoph T Müller-Röver S Audring H: The human hair follicle immune system: cellular composition and immune privilege. *Br J Dermatol.* 2000;142(5):862–873. 10.1046/j.1365-2133.2000.03464.x 10809841

[75] Menni C Kastenmüller G Petersen AK: Metabolomic markers reveal novel pathways of ageing and early development in human populations. *Int J Epidemiol.* 2013;42(4):1111–1119. 10.1093/ije/dyt094 23838602 PMC3781000

[76] Langan EA Philpott MP Kloepper JE: Human hair follicle organ culture: theory, application and perspectives. *Exp Dermatol.* 2015;24(12):903–911. 10.1111/exd.12836 26284830

